# Detection of key transient Cu intermediates in SSZ-13 during NH_3_-SCR deNO_*x*_ by modulation excitation IR spectroscopy†
†Electronic supplementary information (ESI) available. See DOI: 10.1039/c9sc04905c


**DOI:** 10.1039/c9sc04905c

**Published:** 2019-11-18

**Authors:** Alex G. Greenaway, Adrian Marberger, Adam Thetford, Inés Lezcano-González, Miren Agote-Arán, Maarten Nachtegaal, Davide Ferri, Oliver Kröcher, C. Richard A. Catlow, Andrew M. Beale

**Affiliations:** a UK Catalysis Hub , Research Complex at Harwell , Rutherford Appleton Laboratory , Didcot OX11 0FA , UK . Email: andrew.beale@ucl.ac.uk; b Department of Chemistry , 20 Gordon Street , London , WC1H 0AJ , UK; c Paul Scherrer Institut , 5232 Villigen , Switzerland; d Cardiff Catalysis Institute , School of Chemistry , Cardiff University , Main Building, Park Place , Cardiff , CF10 3AT , UK

## Abstract

The small pore zeolite Cu-SSZ-13 is an efficient material for the standard selective catalytic reduction of nitrogen oxides (NO_*x*_) by ammonia (NH_3_). In this work, Cu-SSZ-13 has been studied at 250 °C under high conversion using a modulation excitation approach and analysed with phase sensitive detection (PSD). While the complementary X-ray absorption near edge structure (XANES) spectroscopy measurements showed that the experiments were performed under cyclic Cu^+^/Cu^2+^ redox, Diffuse Reflectance Infrared Fourier Transform Spectroscopy (DRIFTS) experiments provide spectroscopic evidence for previously postulated intermediates Cu–N(=O)–NH_2_ and Cu–NO_3_ in the NH_3_-SCR deNO_*x*_ mechanism and for the role of [Cu^2+^(OH^–^)]^+^. These results therefore help in building towards a more comprehensive understanding of the reaction mechanism which to date has only been postulated *in silico*.

## Abstract 2

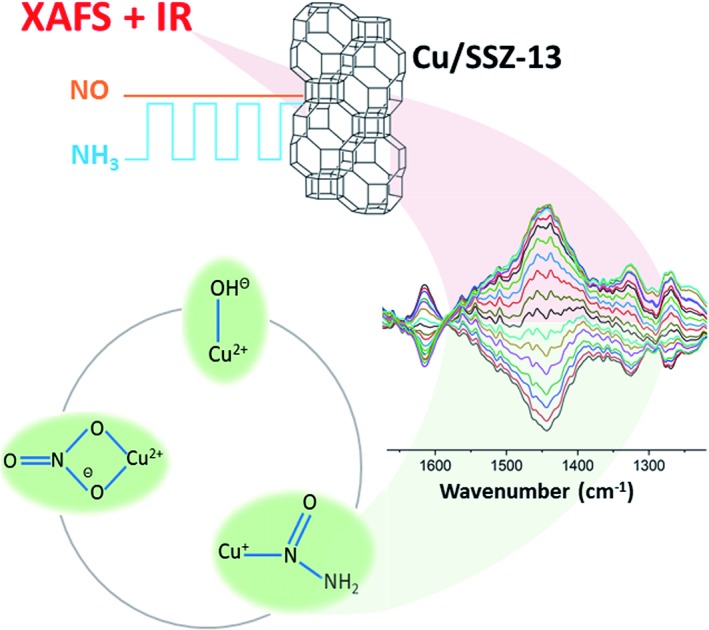
Protagonists in the standard SCR reaction have been caught in the act by modulation excitation IR & XAFS spectroscopy.

## Introduction

Ammonia selective catalytic reduction (NH_3_-SCR) is an efficient way to prevent nitrogen oxide (NO, NO_2_) emissions from heavy duty diesel powered vehicles.^1^–^5^ Cu-exchanged chabazite (in its synthetic form SSZ-13), a small pore zeolite (maximum ring size 8 T-sites), is the subject of a considerable amount of research as it exhibits both a higher activity and selectivity and is less susceptible to (hydro)thermal degradation than other zeolite-based systems (*e.g.* ZSM-5 and beta).^5^–^7^ Mechanistically, the study of NH_3_-SCR is complicated and as of yet, not fully rationalised in terms of reaction kinetics, temperature regimes, and perhaps more relevant here, reaction intermediates. A number of plausible mechanisms have been proposed on the basis of spectroscopic and theoretical data (including works by Paolucci *et al.*, Kwak *et al.*, Janssens *et al.*, Gao *et al.*),^8^–^11^ leading to several points of disagreement regarding a consistent mechanism linking the nature of the catalytically active site to the reaction pathway that enables NO_*x*_ species to be converted to N_2_. *Operando* spectroscopic studies have proven to be successful at providing insight into both active sites and intermediate species for NH_3_-SCR.^12^,^13^ Cu-SSZ-13 has been studied extensively under *operando* conditions with many of the pertinent results outlined in recent reviews.^4^,^12^ For each of the various spectroscopic methods employed, there are limitations when building mechanistic cycles, an example of which is diffuse reflectance infrared Fourier transform spectroscopy (DRIFTS), which has been exploited for several *in situ* NH_3_-SCR studies.^14^–^17^ In this technique, the spectra are dominated by species with large molar extinction coefficients (NH_3_, NH_4_^+^, NO_3_^–^) which renders the detection of short-lived, transient, species difficult. Similarly, for X-ray absorption near edge structure (XANES) spectroscopy, which provides a method to determine the oxidation and coordination state of copper within the sample (*e.g.* Cu^2+^/Cu^+^), it can be challenging to deconvolute signals relevant to the active site from those that originate from other sites in the catalyst that may not be pertinent to the reaction; the large majority of spectroscopic studies are equilibrium-based experiments and many phenomena such as adsorption of reactants, desorption of products and the formation of spectator species occur within similar time frames.^4^ It is thus difficult to rationalise the features that are part of the catalytic process against those that do not correspond to the main reaction pathway. From a mechanistic viewpoint, spectroscopic transient experiments are highly advantageous in studies of NH_3_-SCR catalysts, as demonstrated for V-based and Cu-SSZ-13 catalysts using DRIFTS/UV-Vis and XANES, respectively.^18^,^19^

Concentration modulation excitation (ME) represents an approach to enable the detection of dynamic species directly involved in a reaction,^18^–^20^ which is achieved by removing all parts of the spectroscopic signal from species which are not changing with the same frequency as the modulation excitation frequency. The procedure used in a typical concentration ME experiment is briefly explained here; the theory and data processing required are covered in detail in the ESI† and elsewhere.^20^–^25^

**Fig. 1:**
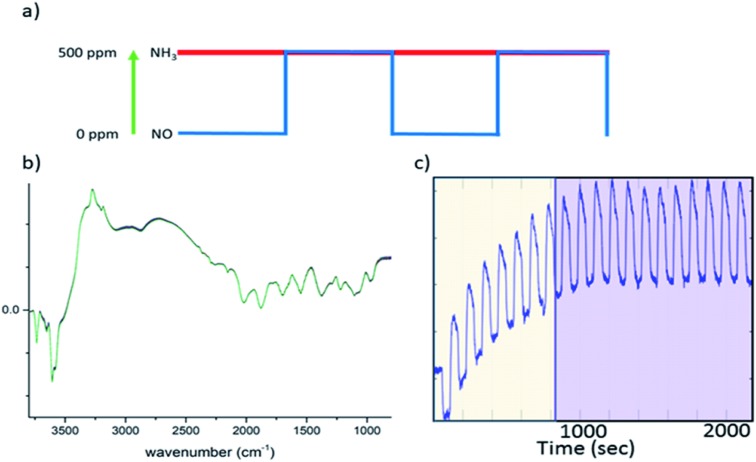
(a) A schematic of a concentration pulse sequence used for the ME experiment, in which NO is turned “on/off” repeatedly between 0 and 500 ppm while the concentration of other reactive components, NH_3_ and O_2_, remains constant. (b) An example of the 2400 DRIFTS spectra collected during this ME experiment (note that there are no significant changes). (c) Time dependent signal at 3655 cm^–1^, demonstrating how the catalyst achieves a quasi-equilibrium state during experiment; the yellow area indicates the cycles where the quasi-equilibrium is not achieved while the purple area highlights the quasi equilibrium region. It is from this latter region that the time-resolved information is derived.

In this study, all ME experiments utilise a pulse sequence which repeatedly stops/starts the flow of NO through the catalyst bed mimicking the standard NH_3_-SCR reaction (see Fig. 1a). Spectra are recorded continuously throughout this work using DRIFTS or XANES in a time-resolved manner in order to follow structural changes. To the eye, these spectra look similar, as the changes between each spectrum are barely discernible (Fig. 1b). After a few cycles of the perturbation, a quasi-equilibrium state is attained, in which the state of the catalyst is considered the same at the start of each cycle (Fig. 1c); these spectra, which correspond to the same point (time) in each cycle, can then be averaged to produce a series of spectra with improved signal-to-noise ratio which are subsequently processed using phase sensitive detection (PSD) (see ESI† for more detail). For this study in particular, features due to strongly adsorbed species (particularly ‘stored’ ammonia), which are not involved in the reaction, are removed.^17^ This also applies to background noise that does not respond to the same frequency as the pulse sequence. The result is a series of spectra with enhanced signal-to-noise ratio providing enhanced sensitivity to species present in the catalytic cycle.

Importantly the concentration modulation excitation DRIFTS data augmented with the application of Density Functional Theory (DFT) allowed us to observe the formation of key intermediates including a copper nitrosamine (Cu–N(=O)–NH_2_) and copper bidentate nitrate species (Cu–NO_3_), while highlighting the regeneration and importance of [Cu^2+^(OH^–^)]^+^ sites in the catalytic process. Ancillary ME XANES experiments confirmed that the measurements were conducted in the presence of the Cu^2+/+^ redox behaviour in the cycle.

## Experimental

### *Operando* ME experiments

#### DRIFTS

Diffuse reflectance Fourier transform infrared (DRIFT) spectra were measured using a Bruker Vertex 70 spectrometer equipped with a Praying Mantis mirror unit (Harrick) and a liquid-N_2_ cooled HgCdTe detector. The homemade spectroscopic cell, based on the commercial Harrick cell but with reduced dead volume, was equipped with a flat CaF_2_ window (*d* = 25 mm; 2 mm thick) and was connected to heated stainless-steel gas supply lines. The outlet of the cell was connected to a FTIR spectrometer (Bruker Alpha equipped with a 70 mm path length gas cell heated to 150 °C). The sieved sample was placed in the sample cup of the cell (57 mm^3^, *ca.* 30 mg). Prior to the experiments, the sample was dried *in situ* in 10 vol% O_2_/N_2_ (100 ml min^–1^) at 400 °C for 2 h. The overall flow was kept at 100 ml min^–1^ (100 000 h^–1^) and the temperature at 250 °C. All DRIFT spectra were obtained by accumulating 10 interferograms at 4 cm^–1^ resolution and 80 kHz scanner velocity (0.9 s per spectrum). During a concentration modulation excitation experiment, solenoid valves were used to automatically switch between gases and were operated by the OPUS software (Bruker). The following pulse sequences were used; 20 cycles of 120 s with 60 s of 500 ppm NO flow on followed by 60 s of NO flow off, into a constant stream of 500 ppm NH_3_, 10 000 ppm O_2_ make up N_2_ (during the “NO off” section of the pulse cycle additional N_2_ was used to keep the total flow constant). The sets of time-resolved DRIFTS spectra obtained from the modulation experiments at equilibrium conditions (Fig. 1c) were time-averaged and subsequently converted into phase-resolved spectra using eqn (S1) (see ESI†). A concentration ME DRIFTS experiment was conducted on an activated sample of Cu-SSZ-13 at 250 °C. The concentration sequence followed 20 cycles in which a 500 ppm flow of NO was added into a stream containing 500 ppm NH_3_, 10 vol% O_2_ and made up with N_2_ for 60 s, followed by 60 s without NO flow (see Fig. 3a and ESI Fig. S17† for out gas analysis).

#### XANES

Cu K-edge XAS studies were performed at the Swiss Light Source (SLS) at the Paul Scherrer Institute, Switzerland, on the SuperXAS beamline.^26^ The polychromatic beam of the 2.9 Tesla superbend was collimated by means of a Si coated mirror at 2.5 mrad, which also was used to reject higher harmonics. Focusing was achieved using a Rh coated toroidal mirror after the monochromator. Measurements were performed by using the Si(111) channel-cut crystal of the quickXAS monochromator. Multiple XAFS spectra were collected at 1 s time resolution. The sieved zeolite sample was placed into a quartz capillary between two quartz wool plugs and sealed in to a gas flow cell. A thermocouple was inserted into the end of the cell such that it reached the centre point of the catalyst bed. The cell was mounted into the beamline so that the beam was focussed towards the front section of the catalyst bed with respect to the gas flow (beam size 0.5 × 0.1 mm). Temperature control was achieved using a hot air blower. The cell was attached to heated gas lines controlled by mass flow controllers (MFCs), the outlet of the gas cell was connected to a Hiden mass spectrometer that was used to follow signals of *m*/*z* 2 (H_2_), 18 (H_2_O), 28 (CO, N_2_), 30 (NO), 32 (O_2_), 44 (N_2_O/CO_2_), and 46 (NO_2_). Prior to the *operando* experiments, the calcined catalysts were subjected to high temperature activation in an O_2_ atmosphere. Samples were heated to 400 °C at 10 °C min^–1^ and held at this temperature until no more changes were observed in the XAS spectra, the sample was then allowed to attain the desired temperature. During the ME experiments the zeolite catalysts were exposed to a similar pulse sequence at 250 °C as outlined above for the DRIFTS experiments, although 10 cycles of 40 s were used in which there was a 20 s NO on pulse followed by 20 s NO off. XANES data processing was performed using IFEFFIT with the Horae package (Athena).^27^,^28^

### Density function theory (DFT) calculations

The VASP code using the Perdew–Burke–Ernzerhof (PBE) functional was employed for the Density Functional Theory simulations. The projector augmented wave (PAW) method with a plane-wave cut-off of 450 eV was used with single *k*-point at the gamma point.^29^–^33^ The structures were optimised with a convergence criterion of 0.02 eV Å^–1^. The converged bulk energies are within 10^–4^ eV. The vibration frequencies were calculated using finite differences to determine the second derivatives. A cube of 8 unit cells of the CHA structure was constructed, which has dimensions of 18.69 Å × 18.69 Å × 18.69 Å containing 94 Si atoms, 2 Al atoms and 192 O atoms. The Cu is placed in the 8 ring as a Cu^2+^ with the framework acting as counter charge due to the acid site hydrogen atoms being removed. In calculations on structures where the Cu^2+^ has been reduced to Cu^+^, an additional NH_4_^+^ cation has been included to maintain charge neutrality for the model.

## Results and discussion

### *Operando* ME DRIFTS experiments

**Fig. 2:**
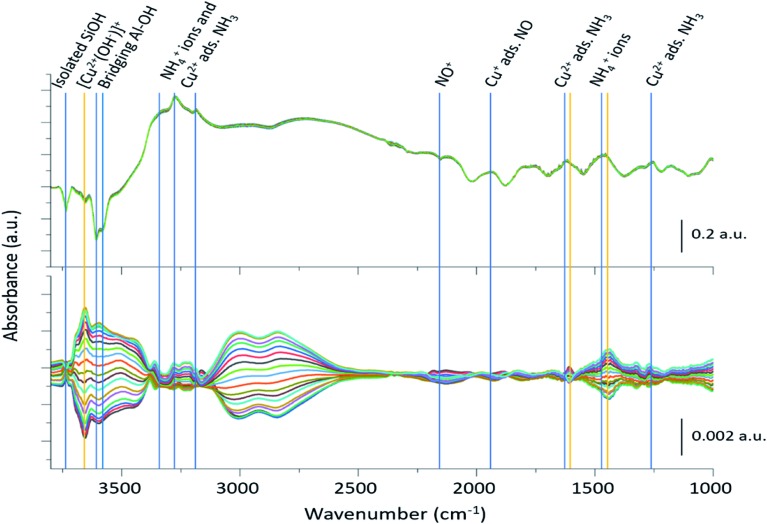
Time-resolved spectra collected during the NO concentration ME DRIFTS experiment (top), and corresponding phase-resolved spectra (bottom). Blue vertical lines indicate features previously reported in the literature (Table 1), whereas orange vertical lines represent features that are enhanced in the phase-resolved spectra, corresponding to newly observed species. The bottom traces indicate how the spectral features are changing with time/gas composition. Note the difference in scale bar units.

The 2400 spectra collected during the ME DRIFTS experiments (Fig. 2, top panel), on visual inspection, appear to be the same, with no clear changes being detected. The spectra display several prominent bands in both the stretching and bending regions, previously identified as vibrations associated with ammonium ions, formed on the zeolite Brønsted acid sites, and NH_3_ coordinated to Cu^2+^ ions (see Table 1 for further assignments). In addition, intense negative bands are seen in the hydroxyl region, corresponding to both Brønsted acidic bridging hydroxyls and silanol groups, along with a weaker feature due to [Cu^2+^(OH^–^)]^+^ centres, showing the formation of adsorbed complexes on these particular sites. The region below 2000 cm^–1^ is, in contrast, dominated by alternating positive–negative signals, attributable to the interaction of NH_3_ molecules, rendering the identification of changes around other features challenging.

**Table 1:** Features observed in the time-resolved spectra of a concentration ME DRIFTS experiment, under SCR conditions (NO gas switch pulse sequence)

Wavenumber (cm^–1^)	Assignment	Ref.
3737	Isolated silanol groups (strong)	17 and 34
3655	[Cu^2+^(OH^–^)]^+^ (very weak)	17 and 34
3602–3588	Brønsted acidic bridging hydroxyls (very strong)	17 and 34
3332, 3182, 1620	Cu^2+^ adsorbed NH_3_ (medium)	15 and 17
3272, 1454	NH_4_^+^ (Brønsted acid site adsorbed NH_3_) (strong)	15 and 17
2158	NO^+^ (very weak)	14
1812	Cu^+^ adsorbed NO (strong)	14
1460	NH_4_^+^/solvating NH_3_	34
1327	Extra framework Al (EFAl) adsorbed NH_3_ (weak)	17
1270	[Cu^2+^(NH_3_)_4_]^2+^ (weak)	17

**Fig. 3:**
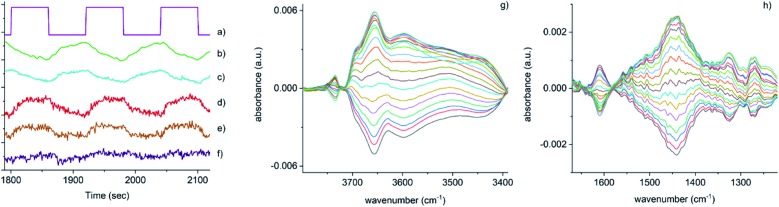
Time-resolved response of several features present in Fig. 2 during the 15–18 cycles of the NO concentration modulation experiment. (a) NO pulse sequence, (b) out-gas NH_3_ integral response (online IR detected), (c) [Cu^2+^(OH^–^)]^+^ integral, (d) 1436 cm^–1^ and (e) 1258 cm^–1^ bands due to the presence of Cu–N(=O)–NH_2_ species, (f) response of the band at 3605 cm^–1^ (Brønsted acid site). Close-up of regions in the phase-resolved spectra of Fig. 2, (g) 3800–3300 cm^–1^ region containing the Brønsted acid site (zeolite OH) and the [Cu^2+^(OH^–^)]^+^ stretches, (h) 1650–1200 cm^–1^ region relevant for NH_3_ coordinated Cu and zeolite.

The corresponding phase-resolved spectra (Fig. 2, bottom panel) reveal how species that are actively involved in the catalytic process evolve over the duration of one complete modulation cycle. These spectra clearly show that most of the contribution of the zeolite framework is removed because it is unresponsive, and that the intense features in the time-resolved spectra do not necessarily correlate to large features in the phase-resolved one. For instance, the relative intensity of the absorption bands of the bridging hydroxyl groups and the [Cu^2+^(OH^–^)]^+^ species is completely reversed. For clarity, the time-resolved response of several of the relevant features and regions of interest in the ME data have been expanded in Fig. 3. Importantly, for completeness, we note that the varying signal of gas-phase NH_3_ measured online to the DRIFTS cell confirms that the ME experiment was performed under actual SCR conditions (Fig. 3b).

Fig. 3 shows that the set of phase-resolved spectra exhibits several features (particularly between 1250 and 1700 cm^–1^) that appear in the time-resolved data, such as NH_4_^+^ ions formed on the Brønsted acid sites and NH_3_ adsorbed on Cu^2+^. In addition, bands at 1812 and 2158 cm^–1^ are also observed that were previously attributed to Cu^+^-NO and NO^+^ (*i.e.* formed by either NO_2_ disproportionation or NO oxidation on Cu^2+^ sites), respectively. It is important to note, however, that the most intense feature in the phase-resolved spectra is that at 3655 cm^–1^, characteristic of [Cu^2+^(OH^–^)]^+^ sites, presenting a larger variation than those corresponding to either silanol groups and bridging hydroxyls. This observation can be attributed to a response of this species to the variation of gas composition, pointing to an active role of this species in the catalytic mechanism, as is clearly illustrated in Fig. 3c, which shows the temporal response of [Cu^2+^(OH^–^)]^+^ species to be affected during the entirety of the NO pulse, which is clear evidence for its consumption and subsequent regeneration under SCR conditions.

A second interesting feature observed in the phase-resolved data concerns a band centred at 1436 cm^–1^, which on first sight appears to be due to NH_4_^+^ species (*i.e.* typically, a combination of both symmetric and asymmetric bending vibrations). Upon closer inspection, this band is clearly red-shifted from the centre of the band due to adsorbed ammonia species, observed in the time-resolved spectra, by approximately 20 cm^–1^. There have been previous studies into the nature and evolution of the broad feature associated with the NH_4_^+^ species, notably by Giordanino *et al.* and Lezcano-Gonzalez *et al.*^17^,^34^ The former study identified the presence of a combination of bands in this region at low temperatures, caused by solvated NH_4_^+^ ions (*i.e.* NH_4_^+^·*n*NH_3_ associations) and the two bending vibrations of NH_4_^+^ ions. Desorption of solvating NH_3_ molecules with increasing temperatures was seen to lead to a gradual intensity decrease and shift to lower wavenumbers of the component at 1463 cm^–1^, and eventually to the appearance of a single broad band at 1430 cm^–1^ due to un-solvated NH_4_^+^ ions attached to the zeolite framework. Lezcano-Gonzalez *et al.* showed that at 250 °C, these NH_4_^+^ ions slowly react under a flow of NO and O_2_, concluding that these species are not an intricate part of the NH_3_-SCR mechanism and more likely remain as a ‘reservoir’ of NH_3_.^17^ Given the time scale over which the (NO pulse) modulation experiment is occurring and the relative intensity of the 1436 cm^–1^ band, it seems unlikely that this is due to the consumption and regeneration of NH_4_^+^ ions. Nevertheless, to further probe the origin of the band at 1436 cm^–1^, a series of additional time-resolved pulse experiments were conducted.

In order to visualize differences in the time response of the bands in the 1400–1530 cm^–1^ region (Fig. S7–S13†) the following pulse sequences were used: (1) adsorption of NH_3_ into a pre-equilibrated catalyst bed of NO and O_2_, and (2) the consequent desorption of NH_3_ (after pre-equilibration of NO, NH_3_ and O_2_). While the features at 1460 and 1436 cm^–1^ share the same response when NO is omitted from the catalyst bed (NH_3_ adsorption under O_2_ flow, Fig. S7 and S8†), clear differences are detected when the catalyst is previously equilibrated with NH_3_, NO and O_2_ (Fig. S9 and S10†), with the intensity of the band at 1436 cm^–1^ increasing more rapidly than the feature at 1460 cm^–1^ upon NH_3_ adsorption. Similarly, a different evolution of these signals is detected after switching off the stream of NH_3_ (Fig. S11 and S12†), with again the band at 1436 cm^–1^ showing a different response from those of the bands more typically observed. This difference in evolutions confirms the 1436 cm^–1^ band to be a separate species to the adsorbed ammonium species typically seen in this part of the spectrum.

**Fig. 4:**
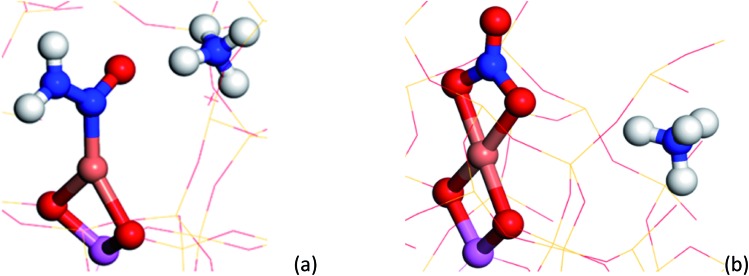
(a) Cu–N(=O)–NH_2_ structure yielded from DFT calculations, containing a N=O and N–N stretches observable at ∼1431 and 1258 cm^–1^ respectively. (b) Bidentate Cu–NO_3_ structure yielded from DFT calculations, containing a N=O stretch at ∼1609 cm^–1^ respectively. The following colour key has been used: Cu (orange), Al (pink), O (red), N (blue), H (white).

To understand the possible origin of the 1436 cm^–1^ band, DFT simulations were performed, focusing on transient species postulated in various reaction mechanisms, but for which there is currently no definitive spectroscopic evidence. The vibrational frequencies of different Cu complexes were calculated, where the active Cu site is placed in the 8 ring as a Cu^2+^ in a cube of 8 unit cells of the CHA structure zeolite (see ESI section† on DFT IR band prediction for further information of all structures calculated and vibrational modes assigned). Of the structures calculated, the closest assignment to such a signal was identified as a Cu nitrosamine (Cu–N(=O)–NH_2_), with an N=O stretching (with some Cu–N component) frequency determined to 1431.2 cm^–1^ (Fig. 4a). In addition to this band, the DFT calculation also predicted a weak band originating from the N–N stretch of the Cu–N(=O)–NH_2_ species at 1258 cm^–1^. Upon (re)inspection of the PSD data there is a weak band in the spectra that evolves and is consumed at exactly the same time (see Fig. 3d and e) and phase angle as the band at 1436 cm^–1^, providing additional support for the presence of a Cu nitrosamine intermediate species. Importantly, Cu–N(=O)–NH_2_ is a key intermediate postulated (on the basis of DFT calculations) in the recent works of Janssens *et al.* and Paolucci *et al.*,^10^,^35^ although this is the first spectroscopic confirmation of its formation and role in the NH_3_-SCR reaction mechanism.

To validate further the findings of the ME DRIFTS experiment, additional modulation experiments were conducted in the absence of NH_3_ (see Fig. S13†). Significantly, these data contain two large features that modulate with the NO pulses; species representative of adsorbed NO (1896 cm^–1^) and NO^+^ (2160 cm^–1^).^16^ The phase-resolved data show neither the consumption and regeneration of [Cu^2+^(OH^–^)]^+^ or any feature close to the signal observed at 1436 cm^–1^ in the experiments described above, thereby supporting the proposal that Cu–N(=O)–NH_2_ intermediate species is part of the SCR catalytic cycle. In addition, the results confirm that the various species observed in the NO pulse ME experiment are consumed and regenerated during the SCR reaction and that they are not spectator species or by-products caused by unselective NO oxidation.

Another feature that shows a strong response to the ME concentration stimulation (NO) is a band centred at *ca.* 1606 cm^–1^. Although the exact origin of this band is unclear, its position falls in the range characteristic of nitrate/nitrite and Cu-amine species, also postulated as relevant intermediates in the SCR mechanism.^10^ We exclude the presence of diamine/tetra-amine Cu species on the basis that although this band could be assigned to an asymmetric bending mode, as can be seen in Fig. 3h, the behaviour of this part of the spectrum does not match at all with that of the 1278 ‘region’ corresponding to the symmetric bending mode of these species.^17^

Examination of possible stable species predicted using DFT suggests a few candidate mono, bidentate nitrate and nitrite species with typical N=O stretching frequencies *ca.* 1595 cm^–1^ (±30 cm^–1^). Of the three structures, the bidentate Cu–NO_3_ structure A (see ESI†) possesses a N=O stretching frequency (*ν*_NO_, *ca.* 1609 cm^–1^) closest to that observed in the phase-resolved IR spectrum. The observation of only one IR active stretch mode (E mode) also implies that the *D*_3h_ symmetry of the NO_3_^–^ molecule is preserved on coordination with the Cu ion; the absence of two bands with similar stretching frequencies (*ν*_as_/*ν*_s_ modes) suggests the absence of a nitrate species of monodentate geometry (*C*_2v_ symmetry). We note, however, that there have been many studies performed on Cu-containing zeolites and that a number of possible nitrates/nitrites have been proposed, including some similar to the structures shown here (particularly Cu–NO_2_, surface adsorbed NO_2_), further suggesting that the accurate assignment of nitrates/nitrites is not trivial.^16^,^36^–^38^ These results also contrast with some observations in which the appearance of ‘Cu-NO_*x*_’ only occurs after the system has moved away from SCR conditions (*i.e.* after NH_3_ flow is switched off).^19^ We note, however, that the Cu nitrate species observed by XAS probably possess a higher Cu–(N)O coordination number or higher number of nitrate ligands than those postulated or observed here. This difference in the nature of the nitrate species may be a consequence of the operating conditions, *i.e.* different temperatures and conversion regimes, but also of their fraction being too short lived for XAS to be detected compared to all other species and thus of the different sensitivity of the two spectroscopic techniques. This observation allows us to conclude that there is probably a difference between the nitrates that play a role in the catalytic cycle as seen here, compared with those that form in the absence of NH_3_ (*i.e.* those that form slowly during NO oxidation).

Additional weak bands are seen in the phase-resolved data of Fig. 2 at 3272 and 3182 cm^–1^, which can be assigned to the stretching modes of both NH_4_^+^ ions and adsorbed NH_3_ on Cu^2+^, respectively, together with a broad feature at 2156 cm^–1^ due to NO^+^.^14^ Finally, two very broad features between 2600 and 3100 cm^–1^ are also seen to be present, which have previously been assigned to hydrogen-bonded coordination spheres around NH_4_^+^ (2800 cm^–1^) and H_3_O^+^ ions (3025 cm^–1^).^39^,^40^ These changes are not unexpected since the Cu ions and zeolite are fully saturated with NH_3_ before the SCR reaction begins with the loss of at least one NH_3_ ligand around the Cu ions necessary for the SCR reaction to take place. The absence of the more commonly observed, yet weaker NH_3_/NH_4_^+^ bending modes in the phase-resolved data suggests that these species undergo only minor modulation during the SCR reaction, which is largely due to their playing an indirect role in the reaction; solvating NH_3_ and NH_4_^+^ species are proposed to provide a reservoir of ammonia for the reaction although as has been shown previously for this catalyst, NH_4_^+^ ions are not particularly reactive.^17^

**Fig. 5:**
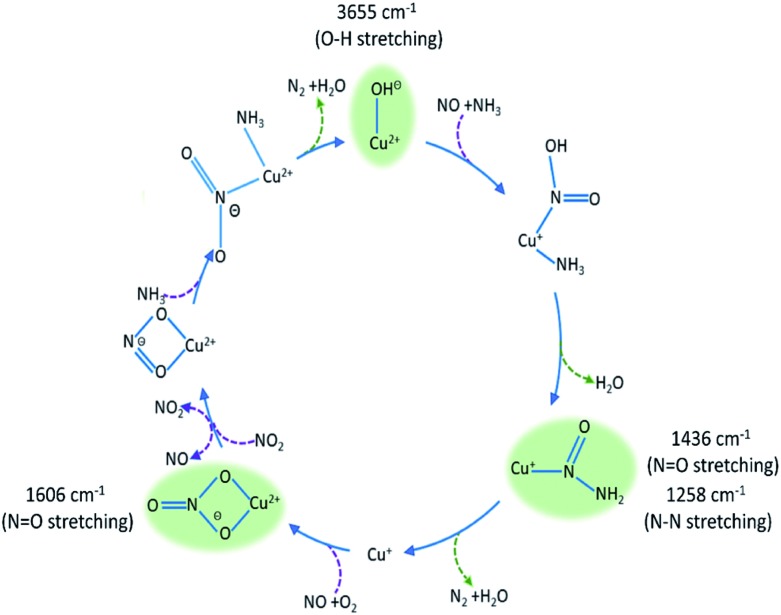
Summary of species evolution during the catalytic cycle, species shaded in green observed in the ME drifts experiments. [Cu^2+^(OH^–^)]^+^ is consumed to make Cu–N(=O)–NH_2_, followed by bidentate nitrate and finally a Cu complex with both NO type and NH_3_ ligands (as anticipated in the mechanism proposed by Janssens *et al.*^10^).

**Fig. 6:**
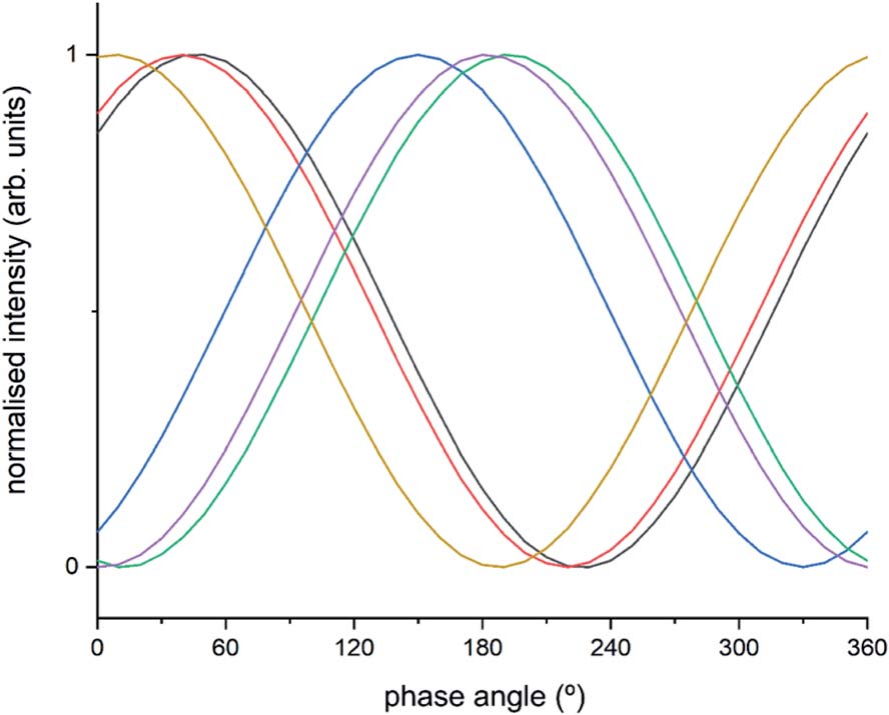
Normalised sinusoidal curves for the respective protagonist IR bands listed in Table S2.† All signals are normalised between 0 and 1, with the response at 3655 cm^–1^ ([Cu^2+^(OH^–^)]^+^) set to 0° phase angle and all other species adjusted accordingly. These species are listed in Table S2† and correspond to band intensity responses due to black = 1258 cm^–1^, red = 1436 cm^–1^, blue = 1606 cm^–1^, green = 2158 cm^–1^, purple = 3182 cm^–1^, orange = 3655 cm^–1^.

The intermediate species seen in this study have previously been proposed as protagonists in the reaction mechanism proposed by Janssens *et al.* (see Fig. 5).^10^ However, analysis of the phase-resolved data, specifically of the in-phase data (*φ*^PSD^ value where signal intensity is highest; Fig. 6) can provide additional insight on the mechanism. Table S2† lists the *φ*^PSD^ values obtained for the signals of the species discussed above. Similar angular values indicate that the species behave kinetically very similarly, while large differences suggest that species are temporally well separated in the chemical process. In the previous work by Janssens *et al.*, it was proposed that the reaction begins with [Cu^2+^(OH^–^)]^+^ and is followed by the formation of Cu–N(=O)–NH_2_.^10^ From Table S2† it appears that both components have a similar phase angle suggesting similar rates and time of formation. Furthermore, the subsequent formation of Cu–NO_3_ followed by NO^+^ (2160 cm^–1^) from the disproportionation of NO_2_, is observed at its maximum intensity with roughly the same phase angle as those associated with Cu–NH_3_ stretch (3182 cm^–1^) which suggests a complex is formed containing both Cu–NH_3_ and NO_2_ towards the end of the cycle. Indeed, if we normalise the phase angle to the start of the catalytic cycle (*i.e.* presence of [Cu^2+^(OH^–^)]^+^ as shown in Fig. 6) it can be seen that there is excellent correlation concerning the order in the phase angle that a species appears and the catalytic cycle shown in Fig. 5.

### *Operando* ME XANES experiments

To probe the oxidation state of the Cu present in the sample under SCR conditions a concentration stimulation ME XANES experiment was conducted on an activated sample of Cu-SSZ-13. The concentration stimulation XANES experiment at the Cu K-edge shows a strong rising absorption edge at 9000 eV due to a 1s–4p dipole transition typical of Cu^2+^, followed by an evolving feature with time attributed to a 1s–4p dipole transition at 8982 eV due Cu^+^.^4^ The phase-resolved data show these two significant features relating to both the Cu^2+^ and Cu^+^ species in the time-resolved data much more clearly. Significantly, the maximum amplitude of the Cu^+^ feature correlates exactly with the minimum amplitude for the Cu^2+^ feature. This experimental observation is a clear indication of the accompanying Cu^2+^/Cu^+^ redox process occurring during the SCR catalytic cycle in these experiments (further details available in ESI Fig. S14†).

### Mechanistic implications of the results

The concentration ME experiments have provided key mechanistic details from both DRIFTS and XANES experiments. The DRIFTS data suggest that the catalytic cycle follows a reaction pathway whereby [Cu^2+^(OH^–^)]^+^ is consumed/reduced to form the transient Cu–N(=O)–NH_2_ species. We note that the NO stretch of this particular species is red-shifted by ∼400 cm^–1^ with respect to where stretches are typically found for heteronuclear N=O_*x*_ species and which, we argue, is caused by electron density removal from the N=O double bond brought about by the formation of an L-type ligand interaction between Cu and N(O) leading to a distribution of 7 electrons over 4 bonds. It is interesting to note that we do not see the initial Cu–NH_3_ or Cu–NO(OH) interactions suggesting that although these must take place they are simply too fast to be observed in this experiment. Indeed, it is well known that Cu^+/2+^–NH_3_ and Cu^+^–NO interactions occur readily, which for NH_3_ even leads to reaction inhibition at low temperatures.^4^ The observation of a sharp feature at 1606 cm^–1^, which could be tentatively attributed to nitrate-type species, and subsequently the appearance of NO^+^ (which has been proposed to be formed through the disproportionation of NO_2_) suggests an important role in the catalytic cycle also for nitrates and nitrites, particularly for completing the Cu redox cycle as has been previously postulated for both NH_3_-SCR in Cu zeolites and observed in hydrogen-promoted hydrocarbon SCR on silver–alumina catalysts.^38^,^41^,^42^ The exact determination of these features is challenging for conventional *operando* DRIFTS experiments as the spectra are dominated by species originating from NH_3_. Interestingly the identification of these intermediates indicates a pathway through which gas phase NO_2_ can be produced in the cycle to circumvent the need to co-feed it to effect fast SCR as is typical in Fe-based systems.^1^ The ME XANES data clearly indicate a Cu^2+^/Cu^+^ redox cycle operating at the same frequency as the concentration pulse. It is interesting to note that previous experiments under steady-state, high-temperature (*i.e.* >250 °C) operational conditions contain no evidence for Cu reduction although it is to be expected that this must occur.^4^ Overall, we find that the observed intermediates have previously been postulated as key species in the catalytic cycle proposed by Janssens *et al.*, although we do note that such species appear in a number of proposed cycles to date.^10^,^43^,^44^

## Summary and conclusions

Cu-SSZ-13 has been studied by concentration ME using both DRIFTS and XANES. The data obtained have provided mechanistic insight into the NH_3_-SCR process on copper containing zeolites. The ME DRIFTS and XANES data reveal the start of the reaction to involve [Cu^2+^(OH^–^)]^+^, highlighting two key intermediates, Cu–N(=O)–NH_2_ and Cu–NO_3_, that would be very difficult to observe using standard *operando* DRIFTS measurements. Using the mechanism in Fig. 5 as a framework to put these observations into a catalytic context, it is possible to argue that these intermediates are perhaps the most significant species in the standard SCR cycle since they lead respectively to the desired product (N_2_), and with the latter species, show how the Cu ion reoxidation occurs. Furthermore, within this mechanism, these species also help to rationalise how NO_2_ is produced circumventing the necessity to utilise additional NO_2_ for effective NH_3_-SCR behaviour. These results, moreover, are in line with predictions from theory, which helps benchmark the ME approach for obtaining potential mechanistic insight importantly obtained under more relevant conditions.^10^

Despite the new insight obtained, we observe that the ME technique is not able to allow us to rationalise fully all the steps in the catalytic mechanism at this temperature or to discriminate definitively between active and spectator species that may evolve at the same rate as the dose response. It may be that better resolution regarding these steps could be realised by performing experiments at lower conversions and/or at lower temperatures. We note, furthermore, that the temperature employed for this study is also unable to discriminate between the low and high temperature mechanism because of the Cu loading, but as it was performed at 250 °C, these results are likely to be more pertinent to a high temperature mechanism; indeed some of the intermediates observed here are different from those recently reported at much lower temperatures (*i.e.* Cu^2+^(NH_3_)_3_(NO_3_)).^4^,^45^,^46^ It is clear, however, that the identification of intermediate states as achieved here can be very useful in guiding the design of the next generation of new and related technologies for deNO_*x*_ applications, perhaps using cationic components that could be more active or at least more environmentally benign or at risk than copper.

## Conflicts of interest

There are no conflicts to declare.

## Supplementary Material

Supplementary information
