# Supplementary material for: Detection of key transient Cu intermediates in SSZ-13 during NH3-SCR deNOx by modulation excitation IR spectroscopy
Source: Chem Sci. 2019 Nov 18;11(2):447–55. doi: 10.1039/c9sc04905c (PMC7067242; doi:10.1039/c9sc04905c)
Supplement: Supplementary file 1 [file SC-011-C9SC04905C-s001.pdf]

Electronic Supplementary Material (ESI) for Chemical Science.
This journal is © The Royal Society of Chemistry 2019

Electronic Supporting Information for: Detection of Key Transient Cu Intermediates in SSZ-13 During NH_3-SCR deNOx by Modulation Excitation IR spectroscopy

Alex G. Greenaway[a,b], Adrian Marberger[c], Adam Thetford[a,b], Ines Lezcano-Gonzalez[a,b], Miren Agote-Aran[a,b], Maarten Nachtegaal[c], Davide Ferri[c], Oliver Kröcher[c], Richard A. Catlow[a,b, d], Andrew M. Beale[a,b]

a.UK Catalysis Hub, Research Complex at Harwell, Rutherford Appleton Laboratory, Didcot OX11 0FA, UK. Email: andrew.beale@ucl.ac.uk

b. UCL, Department of Chemistry, 20 Gordon Street, WC1H 0AJ, UK.

c. Paul Scherrer Institute, Villigen-PSI, Switzerland.

d. Cardiff University, Cardiff, Wales, CF10 3AT, UK

Synthesis, characterisation and catalytic performance

SSZ-13 zeolite (Si/Al=13) was synthesized as described previously, using N,N,N-trimethyladamantammonium hydroxide as structure directing agent in fluoride media, under static hydrothermal conditions. [S1] The proton form of the zeolite is obtained by calcining the sample in air by heating at 1 °C min^{-1} to 120 °C, held for 2.5 h and then at 4 °C min^{-1} to 550 °C, held for 10 h. The copper exchanged form was prepared using a wet ion exchange method in which typically an amount of H-SSZ-13 is added to an aqueous solution of copper sulphate (50 ml of a 0.1 M solution of $CuSO_4$ per gram of zeolite) and heated at 80 °C for 2 h whilst stirring. The product, which consists of well-defined rhombohedral crystals with 2.92 wt % Cu loading, is then recovered by vacuum filtration, washed with copious amounts of water, dried overnight at 80 °C and calcined in air using the procedure outlined previously. Prior to performing *operando* spectroscopic analysis, the zeolites were pressed in to pellets (8 mm bore, 1.5 tonne pressure), crushed and sieved, retaining the 250 – 450 µm fraction for experiments. Note the catalytic performance of these systems has previously been reported. [S1]

PXRD

PXRD patterns were collected to confirm phase purity and crystallinity on a Rigaku Miniflex diffractometer (Cu Kα_1, 1.54056 Å), samples were loaded on to a flat Teflon sample holder. Diffraction patterns were collected between 5.0 and 50.0 ^{o} in 0.02^o steps. PXRD shows that a highly crystalline pure phase of SSZ-13 is present after calcination, subsequent ion-exchange and calcination steps (See Fig. S1). The peak at around 38°, present in the collected diffraction patterns is due to the Teflon liner, peak intensities vary from the simulated data due to preferential orientation and the simulated pattern being from a low Si: Al form containing extra framework K^+ (instead of Cu^{2+}). [S2]

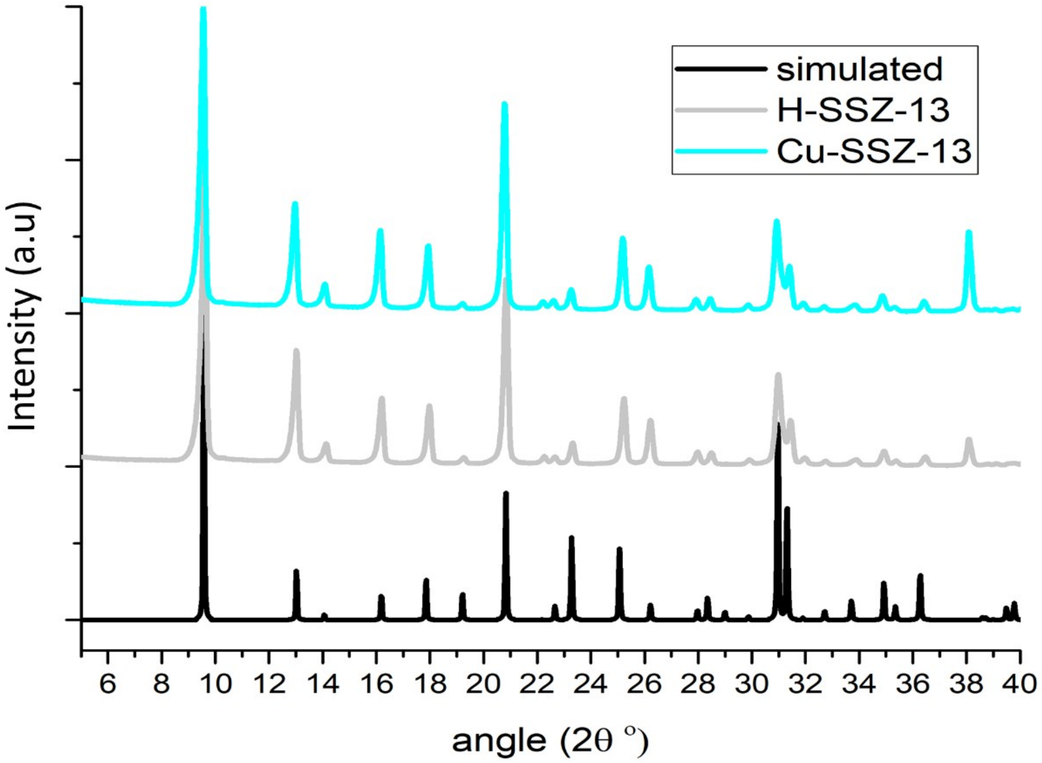

Fig. S1: Simulated PXRD (black), Calcined H-SSZ-13 (grey), Cu-SSZ-13 (light blue), diffraction patterns collected at room temperature in a hydrated state. Note reflection at 38 ° is due to the Teflon sample holder.

SEM and EDX

Catalyst morphology was examined by the Scanning Electron Microscopy (SEM). SEM was carried out using JEOL 6610LV outfitted with a tungsten filament. The acceleration voltage of 20 kV was used. Images were acquired using secondary electron detector. Energy-dispersive X-ray (EDX) analysis was also performed to estimate the Cu weight percent in the catalyst using 80 mm^2 X-Max silicon drift detector from Oxford instruments.

Cu-SSZ-13: - 1 times Copper exchanged and calcined H-SSZ-13 (see Fig. S2a + b)

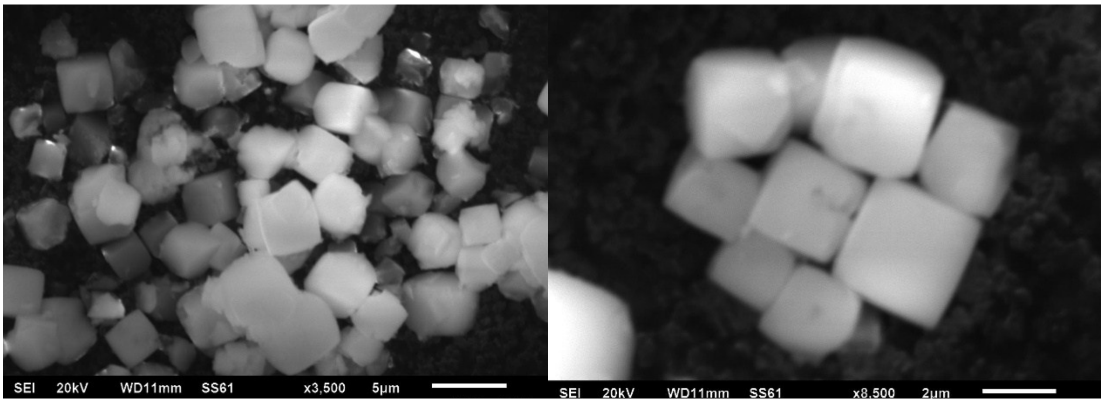

Fig. S2: a) wide angle and b) close up SEM back scattered images showing well defined rhombohedral/cubic crystals with a size distribution between 2 -5 µm.

EDX analysis of Cu-SSZ-13 (see Fig. S3) and composition of materials Table S1: Cu-SSZ-13 has a composition of 2.92 wt % Cu with a Si/Al=13 represents 75 % Cu ion exchanged in to available H^+ sites.

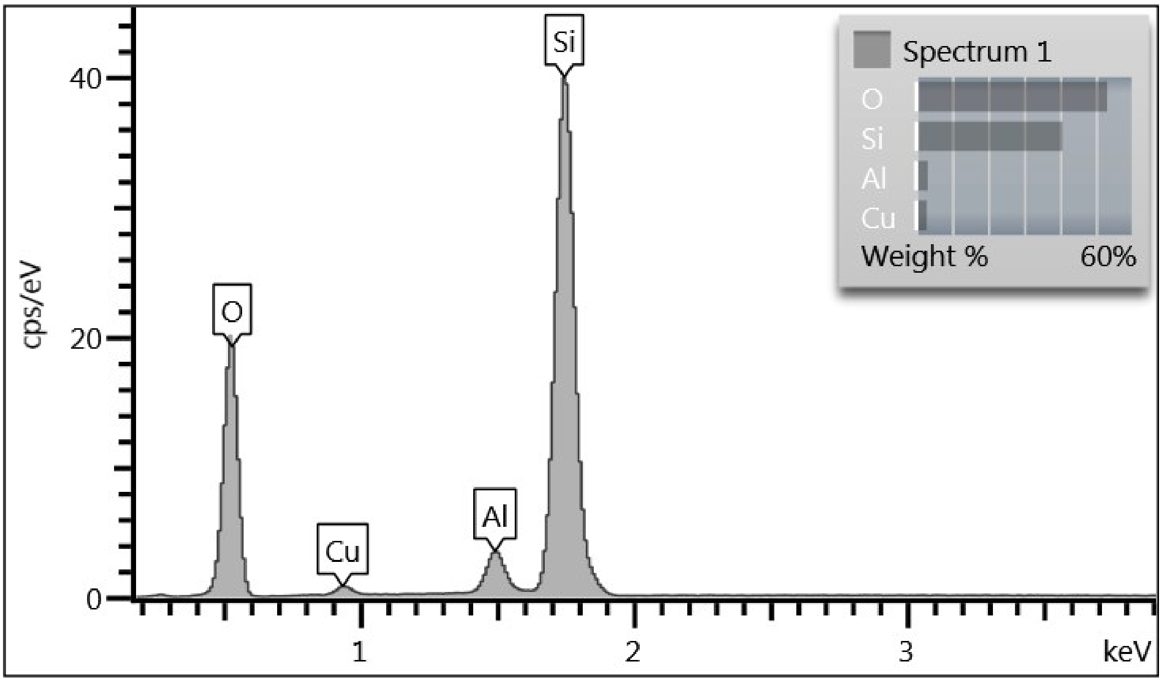

Fig. S3: EDX of Cu-SSZ-13 sample

Table S1: composition analysis of Cu-SSZ-13 sample

Element	Wt%	Wt% Sigma
O	53.12	0.15
Al	3.19	0.04
Si	40.77	0.13
Cu	2.92	0.10
Total:	100.00	

UV-VIS

Diffuse reflectance UV-visible (DRUV) spectra were recorded of samples dehydrated *in situ* on using a Carry 4000 spectrometer (Agilent). All spectra were recorded in the range 200-800 nm and are presented in Kubelka-Munk units, F(R)=(1-R)2/2R, where R is the absolute reflectance of the sampled layer. Samples were loaded into a modified DRIFTS cell and loaded into the spectrometer. Samples were heated at 400 ^oC under flowing oxygen (10 % in N_2) to fully dehydrate the materials (see Fig. S4). Samples were then allowed to attain room temperature, and spectra recorded. The background was subtracted using a dehydrated form of H-SSZ-13. A broad and asymmetric absorption in the UV-Vis spectrum is seen around 16660 cm^{-1} and is due to a ^2E_g and $^2T_{2g}$ transition for a d9 complex in an octahedral crystal field; the asymmetry arising due to the Jahn–Teller distortion be resolved into three sub-components at approximately 20000, 16660 and 13330 cm^{-1} which corresponding to transitions originating from d(xz), d(yz) - d(x^2 - y^2), d(xy) - d(x^2 - y^2) and d(z^2) - d(x^2 - y^2). These features all indicate that there are isolated Cu^{2+} ions present in the dehydrated sample. Importantly the spectra lack other features associated with $[Cu–O–Cu]^{2+}$ dimers and $[Cu2(\mu\text{-}\eta^2{:}\eta^2\text{-}O_2]^{2+}$ dimer which have been previously observed in other materials. [S4]

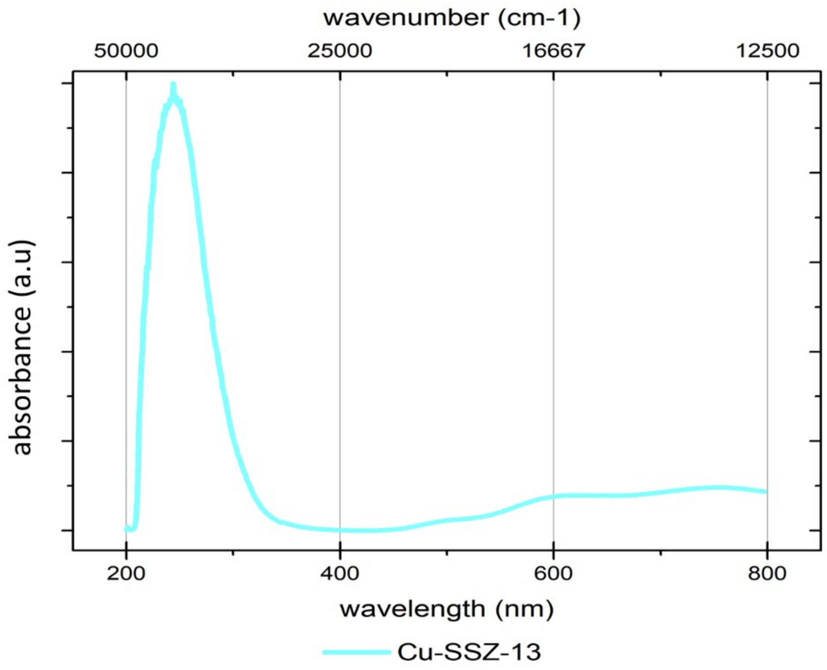

Fig. S4: DRUV spectra collected for Cu-SSZ-13. Samples were collected at room temperature after being activated at 400 ^oC under flowing O_2 (10 %) in N_2.

Modulation Excitation Spectroscopy (MES)

There are now several papers available which described the theory underpinning MES and Phase sensitive demodulation (PSD), however it was initially exploited as a technique to study transient species in heterogeneous catalysis by Baurecht et al. [S5]. Recently a comprehensive review on the technique has been published which demonstrates the broadening application towards heterogeneous catalytic systems by Müller et al. [S6].

The aim of exploiting MES is to enhance a weak signal from a noisy background in a process which is analogous to a digital lock-in amplifier. A requirement of a successful MES experiment is that the studied process is at least quasi-reversible within one modulation period and that the system does not significantly change during multiple cycles over the course of the experiment. The type of modulation used can include any variable that could be altered periodically for example temperature, however in this study all the modulations are based upon concentration stimuli, e.g. a pulse sequence is established in which one of the components is periodically turned on and off (See Fig S5). (It is important to note this simulation takes the form of a square wave excitation, however, these are more commonly used, as they have much simpler setup and require the collection of fewer data sets [S7])

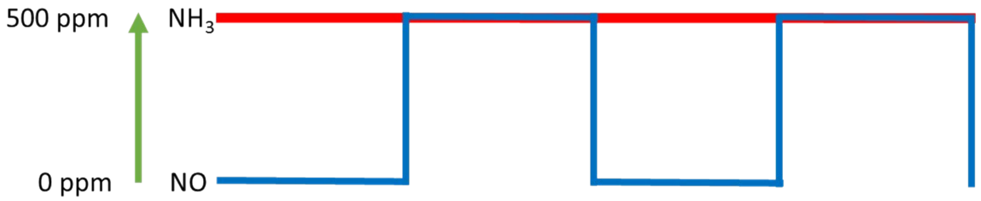

Fig. S5: Pulse sequence showing 2 cycles of a pulse experiment in which NO is periodically turned on and off between 0 and 500 ppm. The total gas flow in ml min^{-1} is conserved by replacing the NO with additional carrier gas when it is turned off.

During a modulation experiment the spectrometer continuously collects data as a series of time-resolved spectra. The species (chemical) present will respond to the stimulation in different ways. For example background noise is not affected by the stimulation and this will appear to be random throughout the pulse sequence, whereas a spectator species may initially increase in concentration as the pulse sequence starts but then may level off and not following the pulse sequence anymore; by spectator species we mean species that adsorb on the catalyst and do not participate further in the reaction pathway or due to a by-product (resulting from a side reaction) that is produced while the catalytic support is still attaining a quasi-equilibrium state. However, it is expected that the concentration of active species which are involved in the catalytic reaction pathway will change in response to the stimulation and as such the relevant signal or feature will also follow the stimulation at the same frequency as the stimulation however it will have a certain time delay depending on its role in the catalytic pathway.

After a few modulation periods the system will reach a quasi-steady state (in which it will return to the same starting point at the start of each cycle), several modulation periods are then averaged to generate a series of time-averaged spectra with enhanced the signal-to-noise ratio. Phase sensitive detection (PSD) is applied to the time averaged spectra to transform them to the phase domain, according to the following equation:

$$I(\varphi^{PSD}) = \frac{2}{T}\int_0^T I(t)\sin(k\omega t + \varphi^{PSD})dt \qquad (S1)$$

where I(t) is the set of time-resolved data, ω the stimulation frequency, k the demodulation index (k = 1 is the fundamental harmonics), T the modulation period and φ^{PSD} the phase angle. Note that in this work we only consider the response of the system where k = 1.

Only signals that follow the fundamental frequency (ω), the active species in the catalytic process, will remain in the phase domain, allowing for discrimination between active and spectator species (which get removed). The signal-to-noise gets enhanced as any noise that is not affected by the stimulation gets eliminated.

φ^{PSD} can be used for the analysis of the studied processes. In the case of a two-step reaction A → B → C, for instance, the characteristic signals of the different species will have maximum amplitudes according to $\varphi_A^{PSD} < \varphi_B^{PSD} < \varphi_C^{PSD}$. Hence, a thorough analysis allows for kinetic differentiation of pathways and lifetimes of active species during the modulation period (see Fig S6).

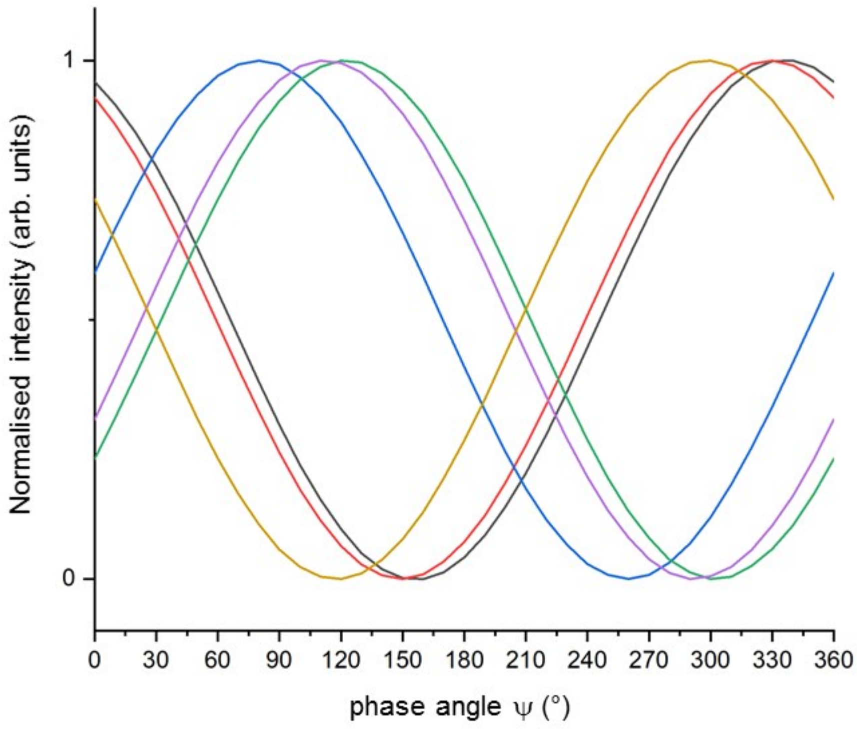

Fig. S6: Normalised sinusoidal curves for the respective protagonist IR bands listed in Table S2. These correspond to black = 1258 cm^{-1}, red = 1436 cm^{-1}, blue = 1606 cm^{-1}, green = 2158 cm^{-1}, purple = 3182 cm^{-1}, orange =3655 cm^{-1}.

Table S2. Phase angle (φ^{PSD}) of the in-phase signal of various relevant species from the NO pulse experiment in NH_3 /O_2 / N_2.

**Wavenumber (cm^{-1})**	**Assignment**	**φ^{PSD} (°)**	**φ^{PSD} (°)**
3655	$[Cu^{2+}(OH^{-})]^{+}$	325	0
3182	Cu^{2+} adsorbed NH_3	110	175
2158	NO^{+}	120	180
1606	Cu-NO_3	80	150
1436	Cu-N(=O)-NH_2	350	30
1258	Cu-N(=O)-NH_2	350	30

Additional DRIFTS experiments

NO, NH_3, and NO / NH_3 adsorption experiments

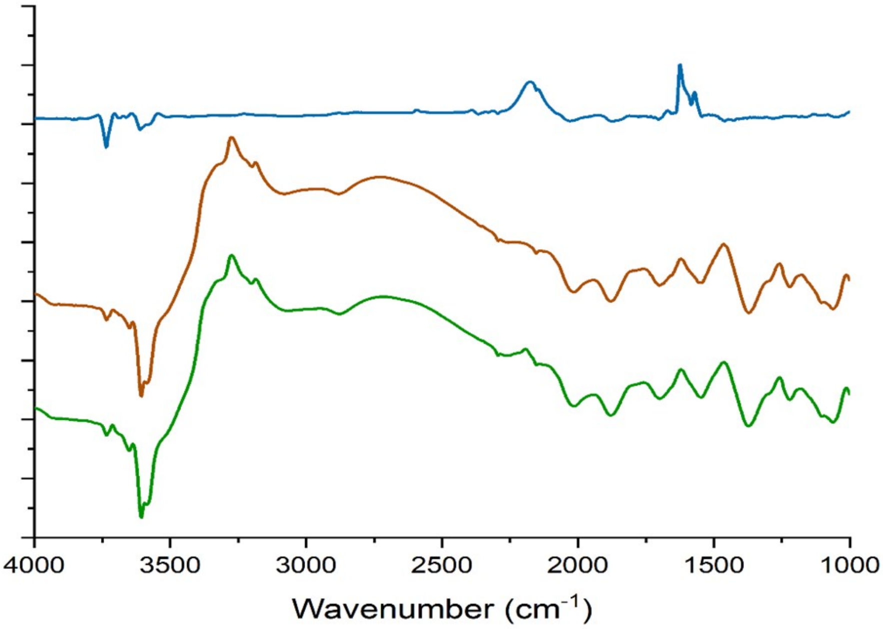

Fig. S7: Spectra attained from steady state DRIFTs experiments of NO (blue trace), NH_3 (brown trace) and NO / NH_3 co-adsorption (green trace).

Table S3:- Major features observed in NO adsorption experiment.

Wavenumber (cm^{-1})	Assignment
3737	Silanol OH
3611	Brønsted acid site
2158	NO^+
1623	Gas phase NO_2/adsorbed NO_3
1562	Gas Phase NO_2/adsorbed NO_3

Table S4:- Major features observed in NH_3 adsorption experiment.

Wavenumber (cm^{-1})	Assignment
3737	Silanol OH
3655	$[Cu^{2+}(OH^-)]^+$
3611	Brønsted acid site
3332	Cu^{2+} adsorbed NH_3
3272	NH_4^+(Brønsted acid site adsorbed NH_3)
3182	Cu^{2+} adsorbed NH_3
1623	Cu^{2+} adsorbed NH_3
1454	NH_4^+(Brønsted acid site adsorbed NH_3)
1270	$[Cu^{2+}(NH_3)_4]^{2+}$

The spectra for the co-adsorption of NO / NH_3 contains many of the same features as the NH_3 adsorption spectra, this is because the spectra acquired are dominated by species with large molar extinction coefficients which are mostly originate from ammonia adsorption (NH_3, NH_4^+). The only significant deviation between the two spectra are features at 2160, 1623 and 1562 cm^{-1}, which are thought to be due to NO oxidation and disproportionation.

NH_3 adsorption in O_2, response from signal between 1400 cm^{-1} and 1530 cm^{-1}

Fig. S8 shows the changes that occur in the DRIFT spectrum of the Cu-SSZ-13 when a stream of NH_3 (500 ppm) is introduced into a bed which has equilibrated under 10 % O_2 mixture made up with N_2. It is clear there is no difference in the evolution between the integral of the 1400-1547 cm^{-1} region, which represents a response for all ammonia/ammonium species vs the integrated area spanning 1400-1459 cm^{-1} area which contains bands due to the presence of NH_4^+ and 1436 cm^{-1} (the centre of the proposed Cu-N(=O)-NH_2 intermediate). In addition, the rates of change of single points at 1436 and 1460 cm^{-1} are also plotted. The 1460 cm^{-1} band was chosen to indicate the formation of NH_3 solvated NH_4^+. The profiles observed share the same rate of change albeit slightly offset; this later offset is due to the formation of NH_3 solvated NH_4^+ occurring only after the initial formation of ammoniated Cu and ammonium ions. NH_3 in O_2 experiment all, i.e. that both the band observed for ammonium ions and those previously assigned to appear at the same rate when exposed to a pre-equilibrated bed.

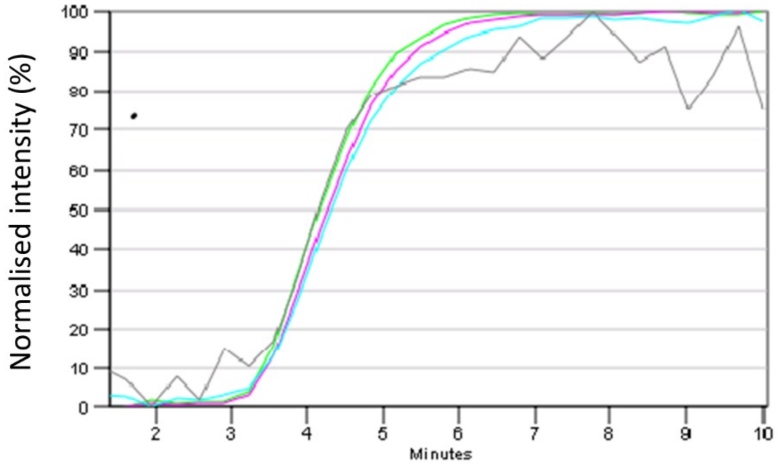

Fig. S8: Response of 1400-1547 cm^{-1} integral (green trace), 1400-1459 cm^{-1} integral (pink trace), 1460 cm^{-1} point intensity (cyan trace) and 1436 cm^{-1} point intensity (grey trace), showing all features share the kinetic response to the introduction of NH_3 in a pre-equilibrated bed of 10 % O_2 and made up in N_2.

NH_3 adsorption in NO

Fig. S9 show the changes that occur to the DRIFTS spectrum of the Cu-SSZ-13 when a stream of NH_3 (500 ppm) is introduced into a bed which has achieved a steady state under a gas stream containing NO (500 ppm)/ O_2 (10%) made up with N_2. Fig. S10 Shows how there is a subtle difference in the kinetics of evolution between the integral of the broad band spanning 1400-1547 cm^{-1}, due to chemisorbed NH_3/NH_4^+, and the integral 1400-1459 cm^{-1} which contains the band observed in the demodulated data which has been assigned to Cu-N(=O)-NH_2 intermediate. This difference in kinetics indicates that the Cu-N(=O)-NH_2 intermediate appears more rapidly than the NH_4^+ species upon the introduction of the NH_3 gas stream and prior to the formation of the formation of NH_4^+ species

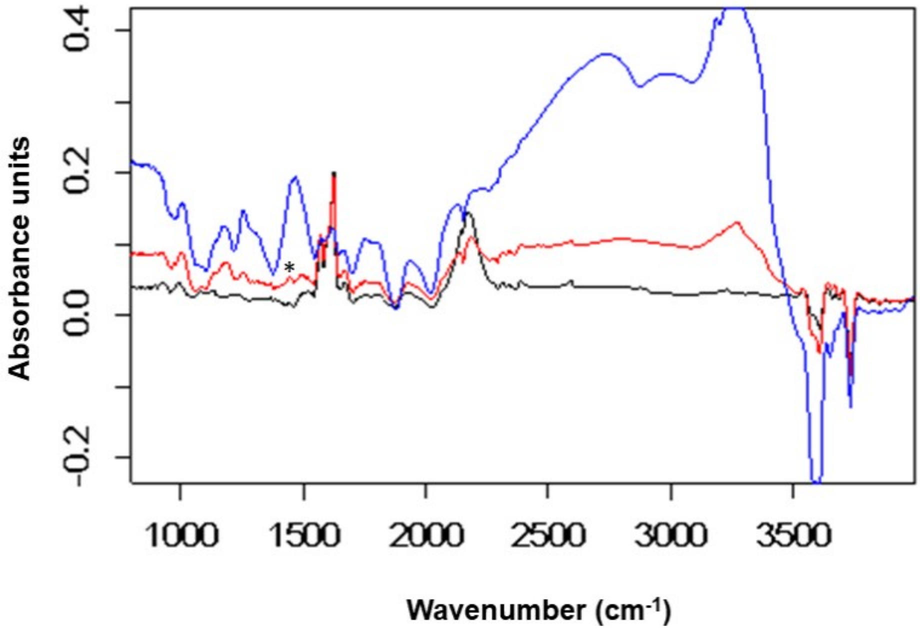

Fig. S9: NO / O_2 / N_2 feed only (black line), after 30 seconds of NH_3 introduction in to NO / O_2 / N_2 gas stream (red line), after approximately 15 minutes NH_3 exposure to NO / O_2 / N_2 gas stream (blue line). Note the weak shoulder in the red trace (highlighted with *), at ~ 1436 cm^{-1}.

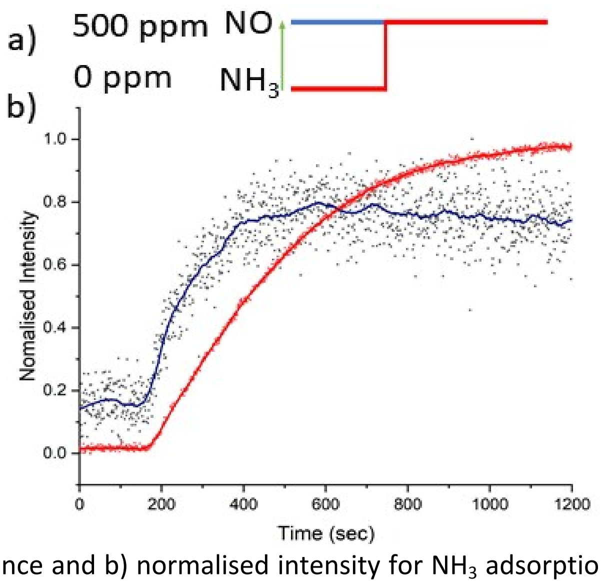

Fig. S10: a) pulse sequence and b) normalised intensity for NH_3 adsorption experiment from NO / O_2 equilibrated catalyst; red line, signal at 1460 cm^{-1}; blue line, signal at 1436 cm^{-1}.

NH_3 desorption in NO

Fig. S11 shows the changes that occur to the DRIFTS spectrum of the Cu-SSZ-13 when a stream of NH_3 (500 ppm) stops flowing over a bed which has achieved a steady state under a gas stream containing NH_3 (500 ppm) NO (500 ppm)/ O_2 (10%) made up with N_2. Fig. S12 reveals significant differences in the kinetics of evolution between the integral of 1400-1547 cm^{-1}, due predominantly to NH_4^+ and the integral 1400-1459 cm^{-1} which represents the peak observed in the demodulated data which has been assigned to Cu-N(=O)-NH_2 intermediate (also present in the point measurement for 1436 cm^{-1}). This difference in kinetics in which the NH_4^+ peak disappears faster than the feature due to Cu-N(=O)-NH_2 intermediate is consistent with a scenario in which the zeolite is acting as a sink for the NH_3, and therefore the material is able to continue to perform catalytic deNO_x for a period of time after the removal of a stream of NH_3, the NH_4^+ is used up during this time while the peak caused by the intermediate remains as there is still enough NH_4^+ / NH_3 present in the zeolite to maintain an environment in which SCR of NO can occur and hence the intermediate Cu-N(=O)-NH_2 is consistently made until the NH_4^+ / NH_3 drops below a level where the deNO_x can be maintained and then the intermediate also decreases in abundance.

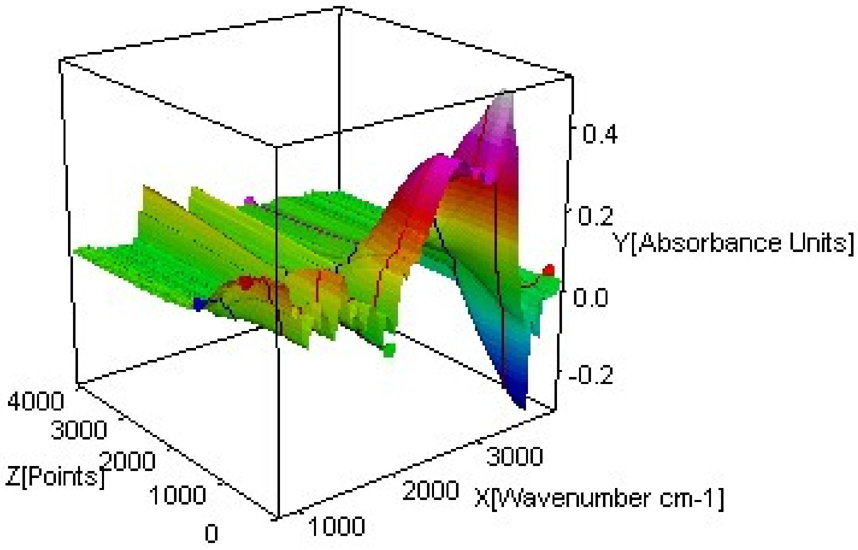

Fig. S11: Gradual evolution of peaks upon the removal of NH_3 from a bed with a steady state conditions of NH_3 (500 ppm) NO (500 ppm)/ O_2 (10%) made up with N_2.

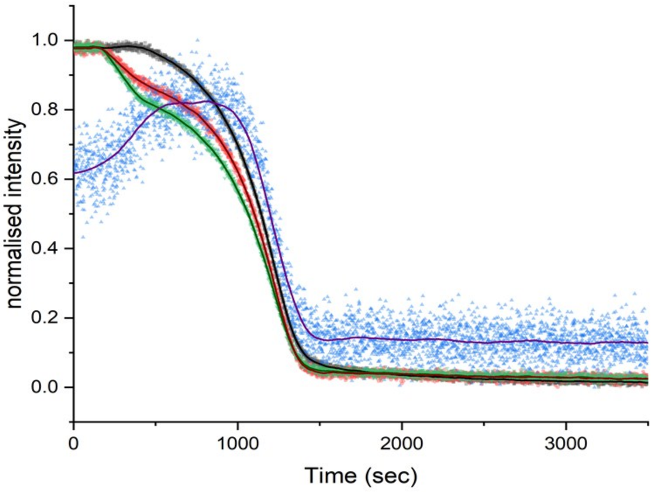

Fig. S12: Normalised intensity for NH_3 desorption experiment from NH_3 /NO /O_2 equilibrated catalyst, ; green line represents integral between 1400-1547 cm^{-1} due to NH_4^+; red line is point intensity at 1460 cm^{-1}; grey line represents integral 1400-1459 cm^{-1} and purple line is point intensity for 1436 cm^{-1}, corresponding to the proposed intermediate.

NO "on / off" modulation experiment

Fig. S13 shows the phase resolved spectra when the following pulse sequences were used; 20 cycles of 120 s with 60 s of 500 ppm NO flow on followed 60 s of NO flow off, into a constant stream of 10 000 ppm O_2 make up N_2 (during the "NO off" section of the pulse cycle additional N_2 was used to keep the total flow the same) over a bed which has achieved a steady state under a gas stream containing O_2 (10 %) made up with N_2. The results show 2 large features that modulate with the NO pulses; species representative of adsorbed NO (1896 cm^{-1}) and by NO^+ (2160 cm^{-1}).

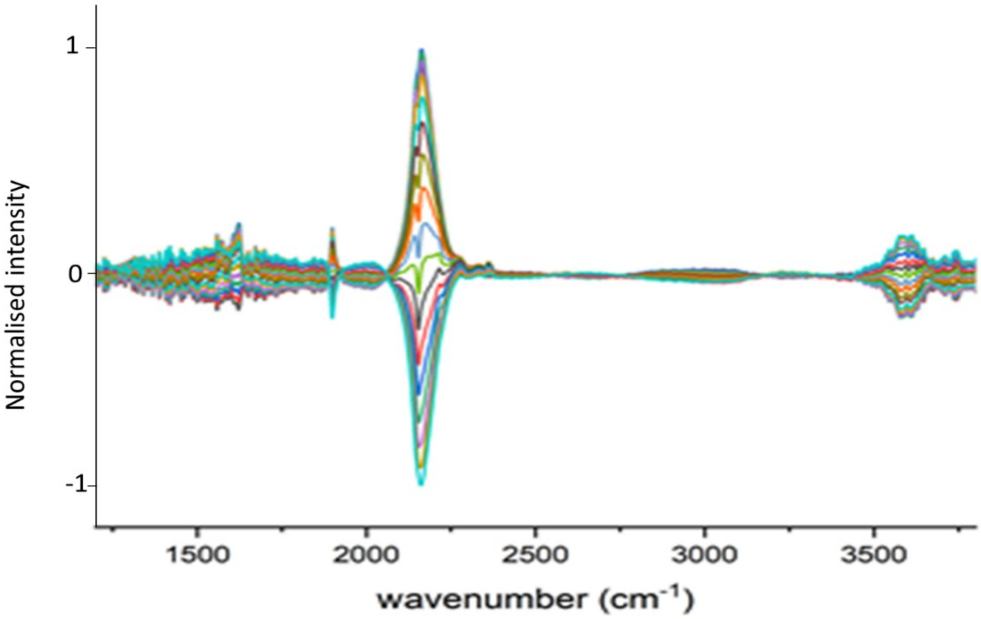

Fig. S13: ME experiment comprising NO pulses in O_2 / N_2. The strong band at 1895 cm^{-1} is ascribed to an NO stretching mode of adsorbed NO and that of 2160 cm^{-1} to adsorbed NO^+.

Gas Hourly Space Calculations

DRIFTS operando experiments

Volume of catalytic bed = 57.3 mm^3

Total flow = 100.00 ml per minute

GHSV = 105263.2 h^{-1}

Gas composition: -

Gas	*Composition (% active gas in N_2)*	*ppm active gas*	*Desired ppm*	*percent total of stream (%)*	*total stream (ml min^{-1})*
NO in N_2	1	10000	500	5	5
NH_3 in N_2	1	10000	500	5	5
O_2	100	1000000	10000	10	10
N_2	100			80	80

XANES operando experiments

Volume of catalytic bed = 13.3 mm^3

Total flow = 19.8 ml per minute

GHSV = 89323.3 h^{-1}

Gas composition: -

Gas	*Composition (% active gas in N_2)*	*ppm active gas*	*Desired ppm*	*percent total of stream (%)*	*total stream (ml min^{-1})*
NO in N_2	1	10000	1262	12.63	2.5
NH_3 in N_2	1	10000	1262	12.63	2.5
O_2	100	1000000	101010	10.1	2
N_2	100			64.7	12.8

Operando MES XANES experiments

The concentration pulse followed 10 cycles in which a 1260 ppm flow of NO is included in to a stream containing 1262 ppm NH3, 10 000 ppm O2 and made up with N2 for 20 s, followed by 20 s where the NO flow was switched off. Gas conditions used: NH_3 (2.5 ml min-1, 1 % in N_2), NO (2.5 ml min^{-1}, 1 % in N_2), O_2 (2 ml min^{-1}, 100 %) and N_2 (12.8 ml min^{-1}) giving a total flow of 19.8 ml min^{-1}, 1262 ppm of NH_3 and NO in 10 vol. % O_2 over a packed bed of 7 mg of zeolite (density of zeolite = 0.526 g cm^3, volume of bed = 0.0133 cm^3). The results obtained from the concentration stimulation MES XANES experiment conducted on an activated sample of Cu-SSZ-13 our displayed in Fig. S14, time-resolved spectra top panel, phase-resolved spectra bottom panel.

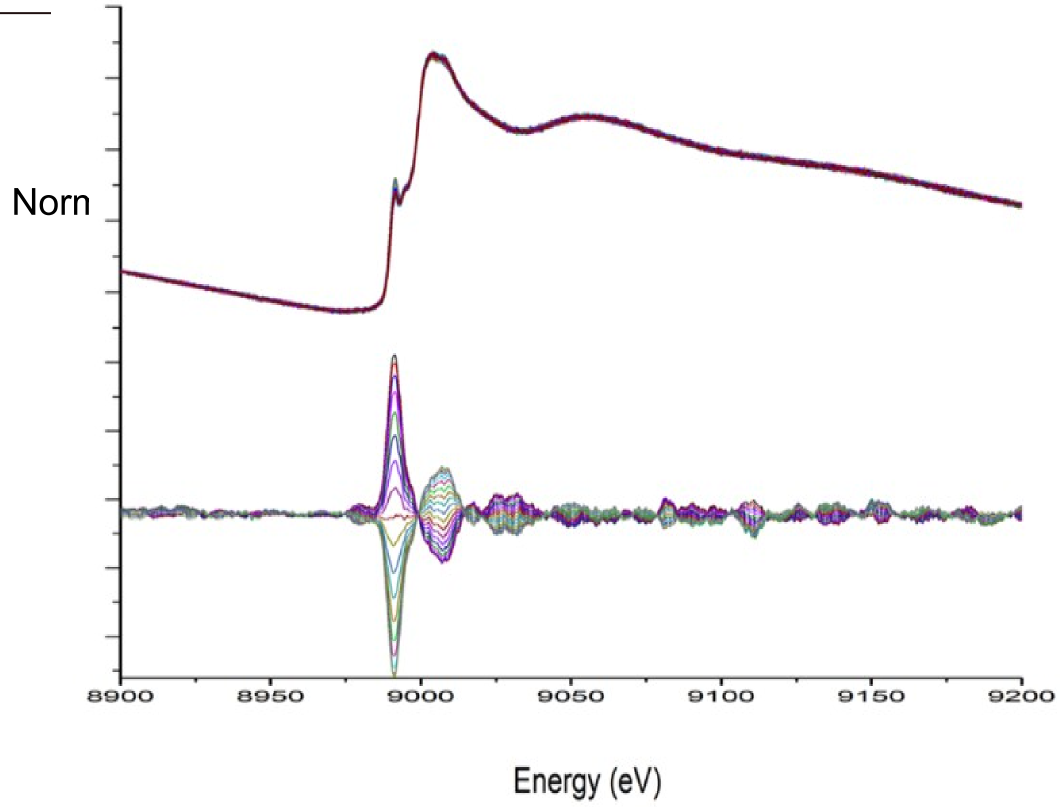

Fig. S14: A concentration stimulation MES XANES experiment on Cu-SSZ-13, (top panel) time resolved spectra, (bottom panel) phase resolved spectra.

XANES Outgas analysis

Mass spec analysis of Cu-SSZ-13, during NH_3 SCR of NO under a concentration stimulation MES experiment with NO ”on/off” (GHSV 90 000 h^{-1}).

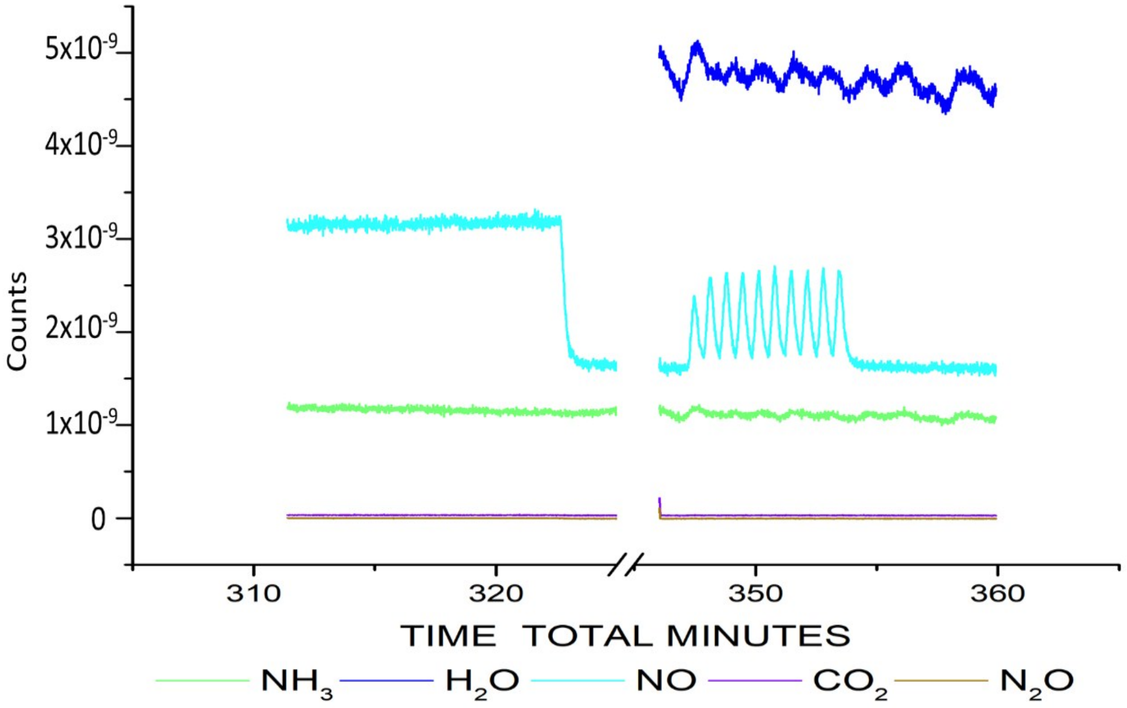

Fig. S15: Mass spec out gas analysis for the concentration stimulation MES experiment with NO ”on/off”. Green) Ammonia, Light Blue) NO, dark blue) Water, purple) CO_2, brown) N_2O. Signal for NO showing clear pulse response to 10 times 20 s on and 20 s off.

FTIR Outgas analysis

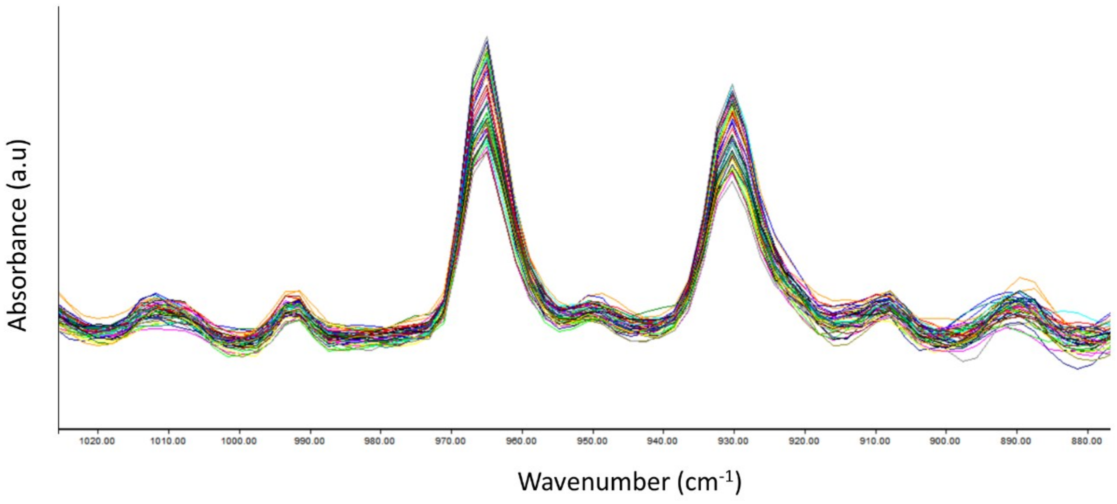

Fig. S16: Gas phase FTIR spectra, showing the response of NH_3 signals across two pulses of a 20 pulse concentration stimulation MES experiment with NO ”on/off”

Prediction of IR bands by DFT

Computational calculations were used to study the origin of the hitherto unassigned spectral features observed in the ME DRIFTS NH_3 SCR deNOx experiments on Cu-SSZ-13. Structures calculated on previously postulated but (spectroscopically) unobserved intermediates in NH_3-SCR literature, [S8, S9, S10, S11] as well as structures based on recent ME DRIFTS experiments on a Vanadia catalyst. [S12]

Computational simulations were completed using Density Functional Theory (employed via the VASP code), using the Perdew-Burke-Ernzerhof (PBE) functional. The projector augmented wave (PAW) method with a planewave cut-off of 450 eV was used with single k-point at the gamma point. The structures were optimised with a convergence criterion of 0.02 eV/A. The converged bulk energies are within 10-4 eV.

Vibration frequencies were calculated using finite differences to determine the second derivatives. A cube of 8 unit cells of the CHA structure zeolite was constructed, which has dimensions of 18.69 Å × 18.69 Å × 18.69 Å containing 94 Si atoms, 2 Al atoms and 192 O atoms.

Cu is placed in the 8 ring as a Cu^{2+} with the framework acting as counter charge due to the acid site hydrogen atoms being removed, in structures for calculations where the Cu^{2+} has been reduced to Cu^{1+} an additional NH_4^+ has been included to maintain charge parity for the model.

Of the structures calculated the Modulation excitation experiments show strong bands associated with 1436 cm^{-1}, of which the DFT structure Cu-N(=O)-NH_2 contains a very similar frequency, this structure was initially postulated by Janssens *et al.* [S11], the frequency is not present in the Cu-(N(=O)-NH_2)-OH species DFT calculation suggesting that the OH has been removed prior to this step in the reaction mechanism, it is postulated therefore that either this species never exists in the reaction pathway or that it is too transient / short lived to be detected in the ME Drifts experiments utilised here. Several structures postulated previously by Kwak *et al.* [S8] (Cu-(N(=O)-OH)-NH_3) and Paolucci *et al.* [S10] (Cu(-NO)(-OH)(-NH_2)) show no calculated frequencies that are observed in the ME experiments, again this is hypothesised to be due to these species never existing in the reaction pathway or that they are too transient to be detected. Regarding nitrates / nitrites the Cu-ONO species postulated by Kwak *et al.* [S8] is not observed, and of the bidentate nitrates calculated Cu-NO_3 (bidentate) structure b shows much better correlation to the observed feature in the ME Drifts experimental data. Finally the Cu derivative of the recently observed transient species in the Vanadia system, Marberger *et al.* [S12] (Cu- N(H_2) -N=O), was found to not be structurally stable during energy minimisation for the Cu based system and so it was not possible to calculate vibrational frequencies for the structure.

Cu-N(=O)-NH_2 species

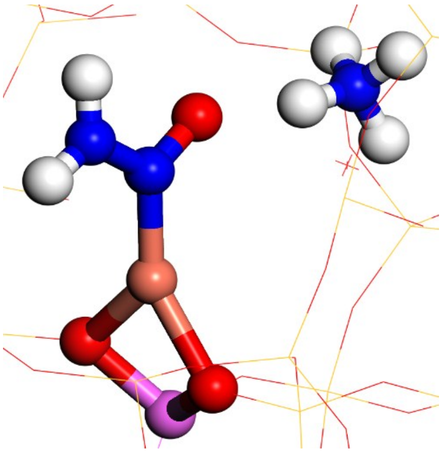

	Wavenumber (cm^{-1})	Description of IR active mode
**1**	3589.26	N-H stretch asymmetric
**2**	3415.354	N-H stretch symmetric
**3**	1553.839	N-H bend
**4**	1431.197	N-O stretch
**5**	1258.279	N-N stretch
**6**	1129.075	N-N stretch
**7**	994.5163	framework
**8**	922.6611	framework
**9**	702.8497	framework
**10**	690.9296	N-N, N-O bend, Cu - N stretch
**11**	685.4527	framework
**12**	668.0429	framework
**13**	657.5581	framework
**14**	651.1161	framework
**15**	641.7644	framework
**16**	606.501	framework
**17**	580.8126	framework
**18**	480.9734	Cu -Al stretch, Cu-O stretch, Cu - N stretch symmetric
**19**	440.0705	Cu -Al stretch, Cu-O stretch, Cu - N stretch asymmetric
**20**	415.2841	Cu -Al stretch, Cu-O stretch, Cu - N stretch symmetric

Cu-(N(=O)-NH_2)-OH

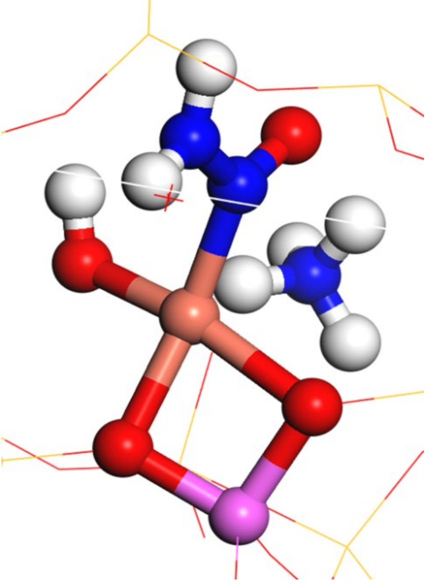

	Wavenumber (cm^{-1})	Description of IR active mode
**1**	3723.962	O-H stretch
**2**	3559.06	N-H stretch asymmetric
**3**	3372.99	N-H stretch symmetric
**4**	1554.554	N-H bend
**5**	1537.459	N-O stretch
**6**	1261.553	N-N stretch
**7**	1116.393	N-N stretch
**8**	936.0451	framework
**9**	879.602	framework
**10**	841.6869	O-H bend
**11**	706.5786	N-N rotation

Cu-(N(=O)-OH)-NH_3

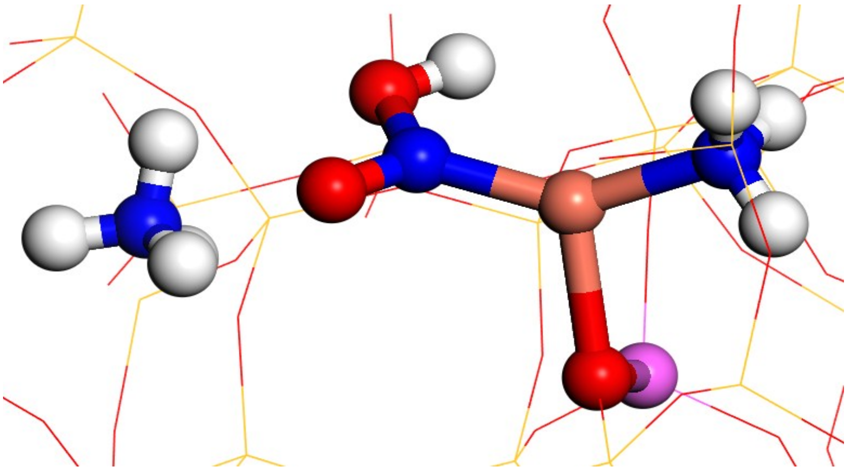

	Wavenumber (cm^{-1})	Description of IR active mode
**1**	3494.717	N-H stretch
**2**	3468.314	N-H stretch
**3**	3359.818	N-H stretch
**4**	3267.169	O-H stretch
**5**	1619.924	N-H bend Cu-NH3
**6**	1612.619	N-O stretch
**7**	1598.073	N-H bend Cu-NH3
**8**	1335.508	O-H bend, weak N-O stretch
**9**	1249.087	N-H bend Cu-NH3
**10**	1054.102	framework
**11**	1009.139	framework
**12**	893.9794	framework
**13**	832.568	N-OH stretch
**14**	812.1672	O-H bend
**15**	742.1576	framework
**16**	735.2519	framework
**17**	703.51	framework

Cu(-NO)(-OH)(-NH_2)

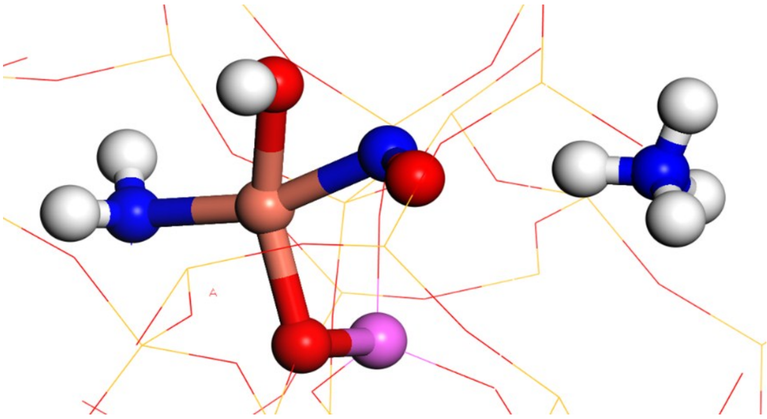

	Wavenumber (cm^{-1})	Description of IR active mode
**1**	3608.688	O-H stretch
**2**	3503.4	N-H stretch
**3**	3486.718	N-H stretch
**4**	3358.652	N-H stretch
**5**	1651.871	N-O stretch
**6**	1620.796	N-H bend
**7**	1615.808	N-H bend
**8**	1211.809	N-H bend
**9**	1070.856	framework
**10**	941.5367	framework
**11**	866.073	O-H bend N-O bend
**12**	727.7907	O-H bend

Cu-NO_2

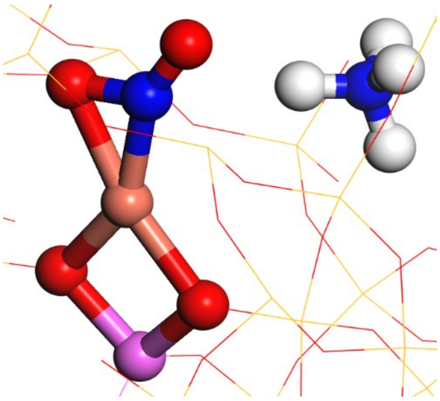

	Wavenumber (cm^{-1})	Description of IR active mode
**1**	1596.631	N-O stretch
**2**	1126.577	N-O stretch
**3**	963.0037	framework
**4**	894.8352	framework
**5**	735.0889	N-O bend
**6**	707.2222	framework

Cu-NO_3 (bidentate) structure *A*

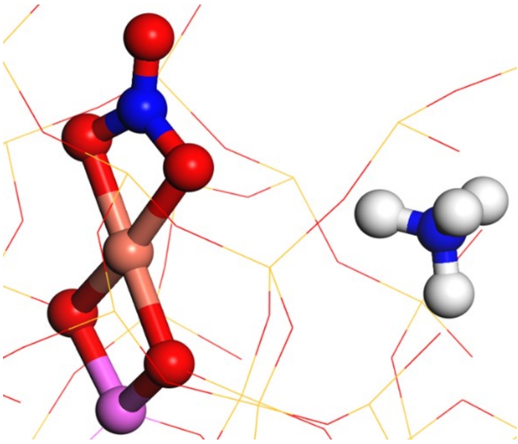

	**Wavenumber (cm^{-1})**	**Description of IR active mode**
**1**	1609.35	free N-O stretch
**2**	1162.575	N-O stretch asymmetric
**3**	989.2475	N-O stretch symmtric, Cu-O bend
**4**	952.9604	framework
**5**	887.7584	framework
**6**	751.5274	N-O bend out of plane
**7**	730.8739	N-O bend Cu-ON stretch
**8**	703.4551	framework

Cu-NO_3 (bidentate) structure *B*

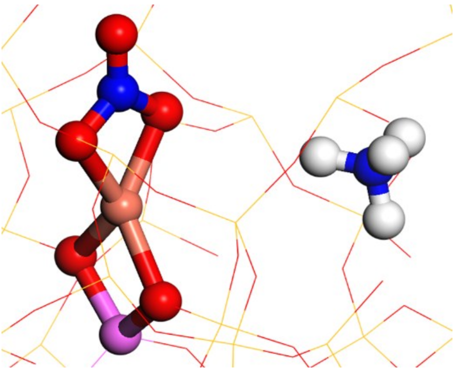

	**Wavenumber (cm^{-1})**	**Description of IR active mode**
**1**	1580.391284	free N-O stretch
**2**	1163.306518	N-O stretch asymmetric
**3**	985.232625	framework
**4**	981.624442	N-O stretch symmetric, Cu-O bend
**5**	915.078511	framework
**6**	734.521256	N-O bend out of plane
**7**	702.400785	N-O bend Cu-ON stretch

References

[S1] Greenaway A.G., Lezcano-Gonzalez I., Agote-Aran M., Gibson E.K., Odarchenko Y., Beale A.M, (2017) *Top. Catal.,* 61, 175-182

[S2] Calligaris, M., Nardin, G., Randaccio L., (1983) *Zeolites*, 3, 205-208

[S3] Giordanino F., Vennestrøm P.N.R., Lundegaard L.F, Stappen F.N., Mossin S., Beato P., Bordiga S., Lamberti C., (2013) *Dalton Trans*., 42, 12741

[S4] Busca G., Lietti L., Ramis G., Berti F., (1998) *App. Catal. B.* 18, 36

[S5] Baurecht, D.; Fringeli, U. P, (2001) *Rev. Sci. Instrum.*, 72, 3782−3792.

[S6] Müller P., Hermans I.,(2017) *Ind. Eng. Chem. Res.,* 56, 1123−1136

[S7] Marchionni V., Ferri D., Kröcher O., Wokaun A., (2017) *Anal. Chem*., 89 (11), 5801–5809

[S8] J. H. Kwak, J. H. Lee, S. D. Burton, A. S. Lipton, C. H. F. Peden, J. Szanyi, (2013) *Angew. Chem. Int. Ed.*, 52, 9985–9989

[S9] M. Iwasaki, in Urea-SCR Technology for deNOx After Treatment of Diesel Exhausts, ed. I. Nova and E. Tronconi, Springer, New York, 2014, ch. 8, pp. 221–246

[S10] C. Paolucci, A. A. Verma, S. A. Bates, V. F. Kispersky, J. T. Miller, R. Gounder, W. N. Delgass, F. H. Ribeiro, W.F.Schneider, (2014) *Angew. Chem. Int.Ed*., 53, 11828–11833

[S11] T. V. W. Janssens, H. Falsig, L. F. Lundegaard, P. N. R. Vennestrøm, S. B. Rasmussen, P.G. Moses, F. Giordanino, E. Borfecchia, K. A. Lomachenko, C. Lamberti, S. Bordiga, A. Godiksen, S. Mossin, P. Beato,(2015) *ACS Catal*., 5, 2832-2845

[S12] A. Marberger, D. Ferri, M. Elsener, O Kröcher, (2016) *Angew. Chem. Int. Ed.*, 55, 11989 –11994

Copies of the raw data can be found at http://tiny.cc/SC-EDG-09-2019-004905
